# Elevated endocan levels are associated with development of renal failure in ARDS patients

**DOI:** 10.1186/2197-425X-3-S1-A264

**Published:** 2015-10-01

**Authors:** L Rahmania, D Orbegozo Cortés, M Irazabal, M Mendoza, C Santacruz, D De Backer, J Creteur, J-L Vincent

**Affiliations:** Department of Intensive Care, Erasme University Hospital, Université Libre de Bruxelles, Brussels, Belgium; Department of Intensive Care, Erasme University Hospital, Université Libre de Bruxelles, Brussels, Belgium

## Intr

Endocan is a proteoglycan preferentially expressed in pulmonary and renal vasculatures and a marker of endothelial dysfunction [1]. Recently, elevated plasma endocan concentrations have been associated with chronic kidney disease stage after renal transplantation [2], but data on acute kidney injury are lacking.

## Objectives

The aim of the study was to determine whether plasma endocan levels were correlated with renal function in patients with acute respiratory distress syndrome (ARDS) and could predict the need for renal replacement therapy (RRT), as an indicator of acute renal dysfunction, during the ICU stay.

## Methods

This was a post hoc analysis of prospectively collected data from 96 consecutive patients with ARDS (Berlin definition) who were not receiving RRT at diagnosis. Plasma endocan concentrations were measured using a quantitative ELISA method (Lunginov, France). We analyzed the predictive value of creatinine and endocan levels at diagnosis on the subsequent need for RRT using the area under the receiver operating characteristic curve (ROC AUC). We dichotomized values of creatinine (using a renal SOFA cut-off of 1.2 mg/dL) and endocan (best sensitivity and specificity based on the ROC curve) to predict need for RRT. All analyses were performed using SPSS 22.0 and a p value < 0.05 was considered as significant. Al values are presented as median with p25-75.

## Results

**Figure 1:**
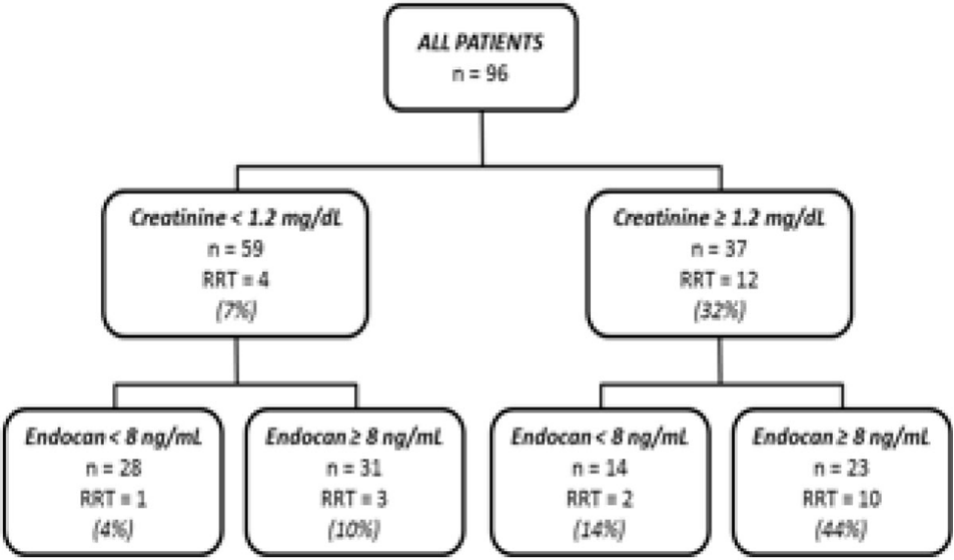
**Need for RRT according to creatinine and endocan.**

Of the 96 patients [APACHE II score 21 (17-27), SOFA score 9 (6-12), creatinine 1.0 (0.7-1.4) mg/dL], 66% had sepsis, 53% needed norepinephrine and 17% needed RRT at some point after diagnosis. Patients who needed RRT had higher APACHE II scores [28(21:30) vs 21(16:26), p = 0.03], higher SOFA scores [12(10:16) vs 8(5:11), p < 0.01], higher blood creatinine levels [1.8(1.2:2.7) vs 0.9(0.7:1.3), p < 0.01] and higher endocan levels [10.0(8.1:30.6) vs 8.1(6.0:10.8), p = 0.02] than those who did not need RRT. ROC AUCs (IC 95%) for creatinine alone, endocan alone or the two together to predict RRT were 0.72 (0.58-0.85), 0.69 (0.53-0.85) and 0.77 (0.64-0.89), respectively. The best ROC AUC cut-off for endocan was 8 ng/mL. Endocan and creatinine values were poorly correlated (r² = 0.052, p = 0.03); when combined they predicted the need for RRT better than did creatinine alone (Figure 1).

## Conclusions

At ARDS diagnosis, elevated endocan levels are associated with subsequent development of renal failure.
